# Hydrogen Permeation Behavior of Locally Reinforced Type IV Hydrogen Storage Vessels

**DOI:** 10.3390/polym18020230

**Published:** 2026-01-15

**Authors:** Guangming Huo, Yu Zhang, Xia Han, Haonan Liu, Xiaoyu Yan, Gai Huang, Ruiqi Li, Shuxin Li, Kaidong Zheng, Hongda Chen

**Affiliations:** 1 State Key Laboratory of Advanced Technology for Materials Synthesis and Processing, Wuhan University of Technology, Wuhan 430070, China; 2 National Energy Key Laboratory for New Hydrogen-Ammonia Energy Technologies, Foshan Xianhu Laboratory, Foshan 528200, China; 3 Wuhan Institute of Marine Electric Propulsion, Wuhan 430064, China

**Keywords:** hydrogen permeation, numerical model, type IV hydrogen storage vessel, local reinforcement

## Abstract

Hydrogen permeation parameters of PA12 were obtained through high-pressure hydrogen permeation experiments conducted under various temperature and pressure conditions. The temperature-dependent mechanism governing the hydrogen permeation behavior of PA12 was further examined using dynamic mechanical analysis (DMA). A multi-field coupled numerical model was established and validated against the experimental results. Based on the validated numerical approach, the hydrogen permeation behavior of a type IV hydrogen storage vessel with local reinforcement was investigated. The results show that both temperature and pressure have a significant influence on the hydrogen permeation performance of PA12. When the temperature is below the glass transition temperature (Tg) of PA12 (48.34 °C), the diffusion coefficient remains low, whereas temperatures above the Tg led to a marked increase in the diffusion coefficient. In addition, the local reinforcement patch effectively prolongs the time required to reach steady-state permeation, reduces the hydrogen permeation flux before and after steady state, and enhances the overall resistance to hydrogen permeation of the type IV vessel. As the diffusion coefficient of the liner material increases, the hydrogen diffusion rate increases substantially, leading to greater hydrogen accumulation in the dome region and higher permeation levels both before and after steady state. These findings provide theoretical guidance and design references for optimizing the hydrogen-resistant performance of type IV hydrogen storage vessels.

## 1. Introduction

Hydrogen storage cylinders, as core equipment in the field of high-pressure hydrogen storage and transportation, serve as key carriers in the implementation of Chinese hydrogen energy strategy and are widely used in hydrogen fuel cell vehicles [1,2].

Among them, type IV hydrogen storage cylinders, composed of a polymer liner, a carbon fiber-reinforced resin-based plastic (CFRP) winding layer, an aluminum (Al) metallic boss, and a valve assembly, offer high hydrogen storage density as well as excellent corrosion resistance and fatigue performance, making them a major focus of current research [3,4,5].

**Figure 1:**
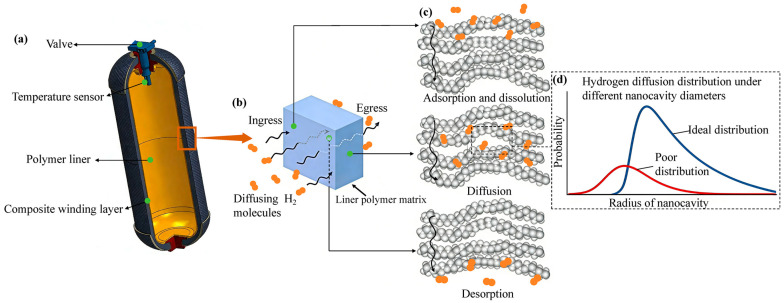
(**a**) Structural diagram of type IV hydrogen storage cylinder; (**b**) Macroscopic diagram of hydrogen diffusion in polymers; (**c**) Microstructure diagram of hydrogen diffusion in polymers; (**d**) Hydrogen distribution under different nanocavity diameters.

In type IV hydrogen storage cylinders, the polymer liner serves primarily as a barrier to prevent hydrogen permeation [6]. However, due to the small molecular size of hydrogen, permeation through the polymer liner is inevitable [7]. The diffusion of hydrogen in polymers is shown in Figure 1 Once permeated, hydrogen can escape along the contact interface between the liner and the composite winding, posing significant safety risks during the operation of fuel cell vehicles, especially in confined environments [8,9]. In addition, during the filling and discharging cycles, hydrogen can diffuse into the polymer liner, creating an internal–external pressure difference that may lead to various forms of damage, such as local buckling, blistering, or even cracking, thereby reducing the service life of the cylinder [10,11,12]. Therefore, understanding the hydrogen permeation behavior of polymer materials is essential for evaluating hydrogen accumulation and for developing optimized designs that mitigate hydrogen-induced damage.

Current analyses of hydrogen permeation characteristics mainly rely on experimental methods [13,14], molecular dynamics simulations [15,16], and numerical analyses [17,18]. Among these approaches, molecular dynamics provides an atomistic framework for investigating the mechanisms of hydrogen adsorption, diffusion, dissolution, and permeation in materials. However, it is challenging to define realistic boundary conditions and parameter settings in such simulations, and the high computational cost limits their direct applicability to engineering material selection. Hydrogen permeation experiments for polymers under high-temperature and high-pressure conditions are generally conducted using the pressure–difference method, which requires stringent sealing performance, high measurement accuracy, and robust safety features, resulting in substantial equipment investment. In contrast, finite element analysis (FEA) can effectively predict the spatial distribution and gradient evolution of hydrogen concentration within materials. Once validated, FEA models can reliably reveal the effects of temperature and pressure on hydrogen permeation behavior and provide practical guidance for engineering applications.

For instance, Zhao et al. [19] employed molecular dynamics simulations to investigate the diffusion behavior of hydrogen in polyethylene (PE). Their results showed that the length of the PE main chain has little influence on hydrogen diffusion, whereas branched and side chains can provide additional diffusion pathways for hydrogen molecules. Qing et al. [20] used molecular simulation software to study the hydrogen permeation behavior and related coefficients of PE and PA, and reported that the hydrogen solubility coefficients of both polymers decrease with increasing temperature, while their diffusion and permeability coefficients increase. Zhang et al. [21] also applied molecular dynamics to examine the hydrogen permeation behavior of high-density polyethylene (HDPE) at different temperatures. Their findings indicated that both the hydrogen solubility and permeability of HDPE increase significantly with temperature. In addition, hydrogen pressure and structural thickness also have notable influences on the hydrogen permeation behavior of polymers. Dong et al. [22] conducted hydrogen permeation tests on HDPE and PA6 under various pressures and observed that the hydrogen permeability of both materials decreases as pressure increases, whereas hydrogen solubility in these polymers is only weakly affected by pressure. Fujiwara et al. [23] further revealed that increasing pressure reduces the free volume within polymers, compressing the diffusion pathways for hydrogen and resulting in lower permeability. From a structural perspective, Hiroyuki et al. [24] analyzed the relationship between crystalline polymer thickness and hydrogen diffusion, showing that the diffusion coefficient of HDPE decreases with increasing thickness due to the extended diffusion path and more pronounced compression of free volume in the amorphous regions. Liebrich et al. [25] reported that due to the insufficient sintering of thin materials and poor melting and consolidation, the diffusion coefficient of laser-sintered PA12 sheets increases with the decrease in thickness. Because thick materials have large pore volumes and contain nanoporous holes, the solubility coefficient increases with increasing thickness. Jan C. et al. [26] conducted hydrogen permeation tests on continuous fiber-reinforced polyamides (e.g., PA6 and PA12) and found that their hydrogen-barrier properties are significantly enhanced compared with those of neat polyamide matrices. Bendine et al. [27] found that strategically placing carbon-fiber strips in the radial and axial directions on the dome of a hydrogen storage cylinder reduced axial and circumferential stresses by 29% and 46%, respectively, under an internal pressure of 70 MPa. Hu et al. [28] further showed that the dome reinforcement (DR) technology can transfer the maximum stress of the Type IV hydrogen storage container to the cross-section of the cylinder, and the smaller the DR Angle, the better the reinforcement effect.

Recently, domestic and international researchers have investigated the hydrogen permeation behavior of HDPE, PA6, and PA11 under various pressure conditions using both molecular dynamics simulations and experimental methods. These studies have provided corresponding material parameters and revealed the influence of material thickness on hydrogen permeation characteristics. However, due to the limited availability of high-temperature, high-pressure hydrogen permeation testing equipment and the incomplete material parameter database for polymers, experimental studies on specialty PA12 liners used in type IV hydrogen storage cylinders, where high barrier performance is essential, remain scarce. Moreover, numerical analyses of the hydrogen permeation behavior of specialty PA12 under multi-field coupled conditions are still insufficient, and the mechanisms governing the effects of temperature and pressure on its permeation behavior have not been clearly elucidated. At the structural level, research on hydrogen permeation in the overall configuration of type IV hydrogen storage cylinders is also lacking, and the accumulation behavior and spatial distribution of hydrogen within the cylinder structure remain to be clarified.

In this study, a combined experimental and finite element approach is employed to investigate the hydrogen permeation behavior of a specialty PA12 material under various temperature and pressure conditions, and to experimentally validate a multi-field coupled numerical method for hydrogen permeation analysis. Based on the validated numerical framework, the hydrogen permeation characteristics of a type IV hydrogen storage cylinder with locally reinforced structures are further examined. The study compares the permeation behavior of cylinders using high-density polyethylene (HDPE), low-density polyethylene (LDPE), and ultra-high-molecular-weight polyethylene (UHMWPE) as liner materials, and evaluates the influence of liner type on the internal hydrogen mass concentration, mass flow rate, and steady-state permeation of the overall type IV cylinder structure.

## 2. Materials and Methods

### 2.1. Materials

To meet the stringent hydrogen-barrier requirements of type IV hydrogen storage vessel liners, a specialized PA12 material was jointly developed with Wanhua Chemical Group Co., Ltd. (Yantai, China). A 400 L plastic liner was manufactured using rotational molding, and circular specimens were subsequently cut from the liner using a custom-designed cutting device in accordance with the GB/T 42610-2023 standard [29]. The PA12 specimens were then placed in a dry, room-temperature chamber, where long-term mechanical compression was applied to relieve the out-of-plane curvature induced by cutting. The final PA12 specimens had a thickness of 4.5 mm and a diameter of 78 mm.

The type IV hydrogen storage vessel was fabricated using T700SC-12000-50C carbon fiber tows (Toray, Tokyo, Japan) and RB240A/B epoxy resin (Swancor Advanced Materials Co., Ltd., Tianjin, Taiwan) for the filament-wound layers. The winding tape had a width of 6 mm and a single-ply thickness of 0.255 mm. A polymer liner was used as the inner container of the vessel. Based on the Automatic Layup Reinforcement technique, prepreg reinforcement patches were bonded to the liner surface in the dome region. The prepreg, supplied by Hengshen Co., Ltd. (Danyang, China), consisted of T700 carbon fibers and epoxy resin, with a resin mass fraction of 35% and a fiber areal weight of 615.77 g/m^2^. Each reinforcement patch had a cross-sectional dimension of 50 mm × 20 mm.

### 2.2. Hydrogen Permeation Testing

The permeability coefficient (P) can be used to reflect the ability of gas molecules to pass through polymer materials. The smaller the p value, the more difficult it is for gas molecules to penetrate the material, resulting in better gas barrier performance of the polymer. The p value is given by [30]:

(1) P = DS

Here, the unit of P is mol·m·m^−2^·s^−1^·Pa^−1^. S is the solubility coefficient, (mol·m^−3^·Pa^−1^), representing the thermodynamic properties between gas molecules and polymers, The D is the diffusion coefficient, (m^2^·s^−1^), which represents the dynamic characteristics between gas molecules and polymers.

**Figure 2:**
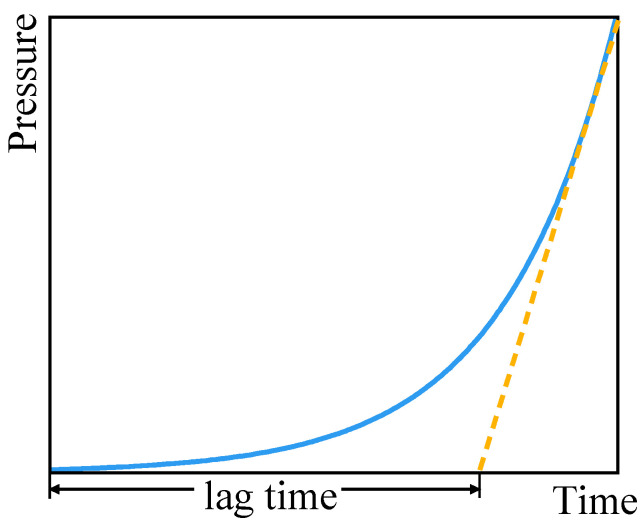
Schematic diagram of the lag time calculation method.

Under high vacuum conditions, the D value is calculated by the lag time when the equilibrium state is reached [31]. The calculation formula is as follows:

(2) $$D\;=\;\frac{L^{2}}{{6\theta }_{0}}$$

where L represents the thickness of the sample, (m). $\theta_{0}$ represents the lag time. The principle of the lag-time method is shown in Figure 2. In the fitting diagram, the pressure and time are in a linear part. The distance from the intersection points of the fitting straight line with the *X*-axis to X=0 represents the lag time. During this period, the gas undergoes non-steady-state diffusion in the material.

**Figure 3:**
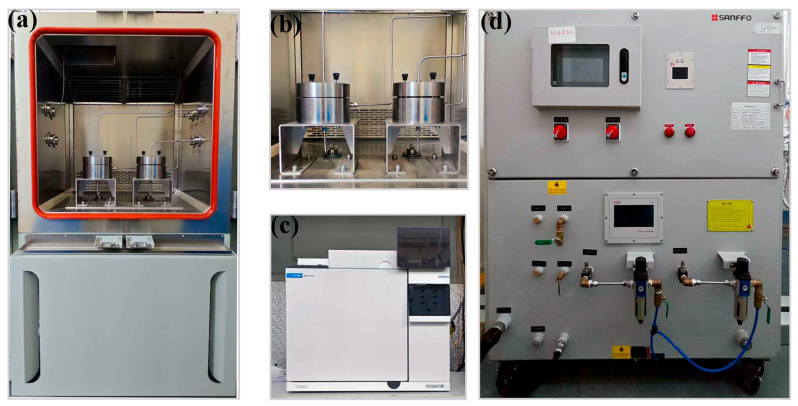
The hydrogen permeation test platform: (**a**) hydrogen permeation environmental chamber; (**b**) gas permeation analyzer; (**c**) gas chromatograph; (**d**) high and low temperature mold temperature controller.

Hydrogen permeation tests were conducted using a customized permeation apparatus (MTU-S-900-E-MP, MIXMATOR, Shanghai, China), as shown in Figure 3. During the experiment, the specimen was first mounted between the high-pressure and low-pressure chambers. High-purity nitrogen was then used to purge the high-pressure side, the low-pressure side, and the entire gas line to ensure that the oxygen and moisture levels met the experimental requirements. Subsequently, a temperature-control unit (TEM-620W-CT4, MYPET, Shanghai, China) was employed to provide a constant thermal environment, as illustrated in Figure 3d. After the system reached the required stability for sampling, the gas composition on the low-pressure side was continuously monitored and quantitatively analyzed using a gas chromatograph (8890-G3540A, Agilent Technologies, Santa Clara, CA, USA). The hydrogen permeation coefficient (*P*), diffusion coefficient (*D*), and solubility coefficient (*S*) of the specialized PA12 material were measured at two temperatures (15 °C and 55 °C) under hydrogen pressures of 7 MPa and 70 MPa, respectively.

### 2.3. Results of the Hydrogen Permeation Experiments

**Figure 4:**
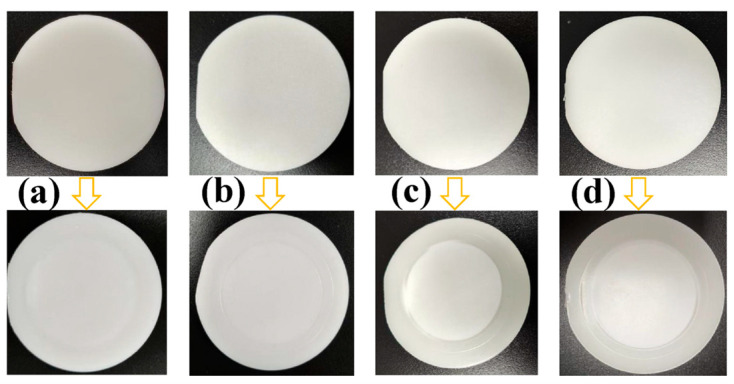
PA12 samples before and after hydrogen permeation testing: (**a**) 7 MPa-15 °C; (**b**) 7 MPa-55 °C; (**c**) 70 MPa-15 °C; (**d**) 70 MPa-55 °C.

Figure 4 shows the PA12 specimens tested under different conditions. For each test condition, five valid specimens were evaluated, and the reported results represent the average values of these five specimens. The hydrogen permeation results of PA12 at various temperatures and pressures are summarized in Table 1. At 15 °C, when the pressure increases from 7 MPa to 70 MPa, the hydrogen permeability coefficient significantly decreases from 1.31 × 10^−15^ to 1.68 × 10^−16^ mol·m·m^−2^·s^−1^·Pa^−1^, corresponding to a reduction of 87.18%. At 55 °C, increasing the pressure from 7 MPa to 70 MPa reduces the permeability coefficient from 2.02 × 10^−15^ to 4.60 × 10^−16^ mol·m·m^−2^·s^−1^·Pa^−1^, a decrease of 77.23%. This behavior is mainly attributed to the compression of polymer chain segments under higher pressure, which reduces the free volume and narrows the migration pathways for hydrogen atoms, thereby lowering the diffusion coefficient.

The influence of temperature on the hydrogen permeation resistance of the material is significant. At the same pressure, the hydrogen permeability coefficient at 55 °C is higher than that at 15 °C. When the temperature increases from 15 °C to 55 °C, the hydrogen permeability coefficients at 7 MPa and 70 MPa increase by 54.20% and 173.81%, respectively. Therefore, the effect of raising the temperature from 15 °C to 55 °C on the hydrogen permeability coefficient is more pronounced than that of increasing the pressure from 7 MPa to 70 MPa.

**Figure 5:**
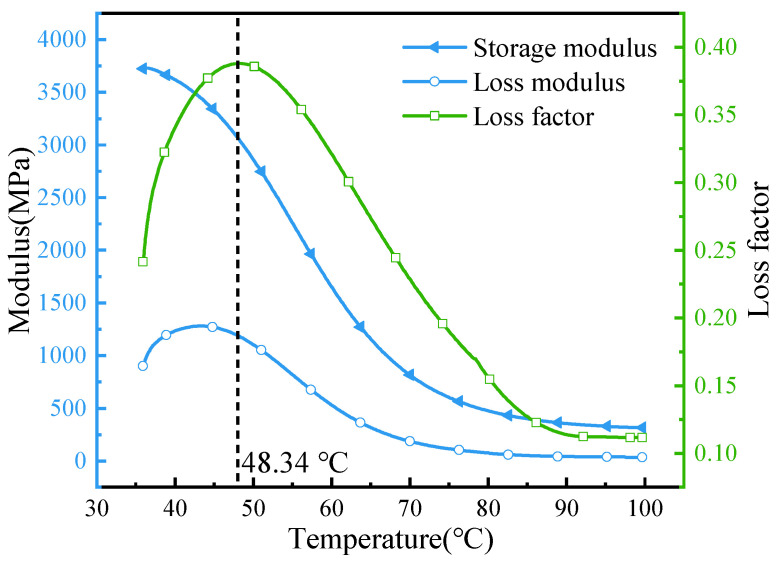
Time-dependent curve of the dynamic mechanical properties of PA12.

To clarify the differences in hydrogen permeation behavior of PA12 at different temperatures, the thermomechanical properties of PA12 were analyzed using DMA. Rectangular PA12 specimens with dimensions of 35 mm × 10 mm × 4 mm were mounted on aluminum clamps and heated from 30 °C to 100 °C at a constant rate of 3 °C/min under a tensile dynamic load of 1 Hz. During the test, the storage modulus ($E^{\prime }$), loss modulus ($E^{\prime \prime }$), and loss factor (tan⋅δ) were recorded as functions of temperature, as illustrated in Figure 5. It can be seen that a distinct and sharp peak appears on the tan⋅δ curve at 48.34 °C. This peak corresponds to the glass transition temperature (Tg) of the PA12 material, which is determined to be 48.34 °C.

Since 15 °C is below the Tg of the specialized PA12, the chain segments in the amorphous regions possess insufficient thermal energy and can only undergo limited local vibrations, while long-range cooperative motions are frozen. As a result, hydrogen molecules must overcome a higher energy barrier to migrate through the amorphous phase, and the diffusion coefficient is significantly reduced due to poorly connected transport pathways. Meanwhile, in the glassy state, the intermolecular van der Waals and hydrogen-bond interactions are stronger, restricting the mobility of polar sites such as amide groups. Their reduced accessibility further hinders the insertion of hydrogen molecules between adjacent chains [32,33]. When the temperature increases from 15 °C to 55 °C, the amorphous chain segments gain sufficient energy to activate internal rotations and enhance long-range cooperative motions, thereby improving the connectivity of transport pathways. The higher temperature also increases the collision probability between hydrogen molecules and polar sites, promoting hydrogen dissolution and resulting in an overall decrease in hydrogen-barrier performance [34,35]. Therefore, under identical pressure conditions, PA12 exhibits superior hydrogen-permeation resistance at 15 °C compared with 55 °C.

## 3. Theoretical and Numerical Analysis Methods

### 3.1. Hydrogen Permeation Theory

The permeation behavior of hydrogen in PA12 can be described using the dissolution–diffusion theory proposed by Calvet and Klopper M.H. [36,37]. The diffusion of hydrogen is driven by the gradient of chemical potential, and under isothermal conditions, the chemical potential μ and chemical potential gradient ∇μ can be expressed as shown in Equations (3) and (4) [38]:

(3) $$\mu =\;\mu^{0}\;+\;\mathrm{RTlnC}\;-V_{H}\sigma_{H}$$

(4) $$\nabla \mu =\mathrm{RT}\frac{\nabla C}{C}-V_{H}\nabla \sigma_{H}$$

where $\mu^{0}$ is the chemical potential at the standard state (J/mol). R represents the universal gas constant, 8.314 J/(mol·K). T donates the temperature (K). ∇ denotes the gradient operator. C is the hydrogen concentration (kg/m^3^). $V_{H}$ is the partial molar volume of hydrogen in the polymer (m^3^/mol) [39]. $\sigma_{H}$ represents the hydrostatic pressure effect, defined as the average of the three normal stress components, which can be expressed as:

(5) $$V_{H}\;=\;V_{s}\frac{\partial }{\partial C}\left(\frac{\Delta V}{V_{0}}\;+{\;\beta }_{T,p}\right)$$

(6) $$\sigma_{H}=\frac{1}{3}\sum_{i}^{3}\sigma_{\mathrm{ii}}=\frac{\sigma_{11}+{\;\sigma }_{22}+{\;\sigma }_{33}}{3}$$

Here, $V_{s}$ is the molar volume of an ideal gas under standard conditions (m^3^/mol). $V_{0}$ is the polymer volume at zero pressure without hydrogen (m^3^). ΔV represents the volume of gas permeating into the polymer (m^3^). $\sigma_{11}$, $\sigma_{22}$ and $\sigma_{33}$ denote the three normal stress components (Pa).

The hydrogen diffusion flux J is proportional to the gradient of the chemical potential ∇μ, as described in Equation (7):

(7) $$J=-\frac{\mathrm{DC}}{\mathrm{RT}}(\frac{\nabla C}{C}\mathrm{RT}-V_{H}\nabla \sigma_{H})$$

Here, D denotes the hydrogen diffusion coefficient (m^2^/s). The transport of hydrogen molecules in the permeation system follows the law of mass conservation. In this study, the migration of hydrogen within the vessel material is described using the mass conservation of the diffusing phase, as expressed in Equation (8):

(8) $$\frac{\partial }{\partial t}\int_{V}(C)\mathrm{dV}\;+\int_{s}J\cdot \vec{n}dS\;=\;0$$

where V denotes an arbitrary diffusion volume (m^3^). t is the time (s). S represents the boundary surface area of volume V (m^3^). $\vec{n}$ is the outward unit normal vector on surface S. By applying the divergence theorem, the final governing equation of the hydrogen diffusion model can be expressed as:

(9) $$\frac{\partial C}{\partial t}-\nabla \cdot \left(D\nabla C\right)-\nabla \cdot \left(\frac{DCV_{H}}{\mathrm{RT}}\nabla \sigma_{H}\right)\;=\;0$$

### 3.2. Simulation Method

The heat transfer process and hydrogen diffusion in materials exhibit strong similarities in terms of governing equations, transport mechanisms, and corresponding physical quantities, demonstrating a one-to-one correspondence between the two phenomena [40]. According to Fourier’s law, the heat flux q, defined as the amount of heat transferred per unit area per unit time, is given by:

(10) q = −k∇T

Here, q denotes the heat flux (W/m^2^). T is the temperature (K). k represents the thermal conductivity (W/(m·K)). And ∇ is the gradient operator. Assuming that no internal heat source exists and the conservation of energy holds, Equation (10) can be rewritten as Equation (11):

(11) $$\rho C_{p}\frac{\partial T}{\partial t}\;+\;\nabla \cdot q\;=\;0$$

where ρ is the density (kg/m^3^), $C_{p}$ is the specific heat capacity (J/(kg·K)), and t denotes time (s). When ∇k = 0, combining Equations (10) and (11) yields Equation (12).

(12) $$\frac{\partial T}{\partial t}=a\nabla^{2}T$$

Here, α is the thermal diffusivity (m^2^/s).

The diffusion flux corresponding to the heat flux density represents the number of diffusing species passing through a unit area per unit time, and is given by:

(13) J =−D∇C

Here, J is the diffusion flux (kg/(m^2^·s)). D is the diffusion coefficient (m^2^/s). C is the mass concentration (kg/m^3^). Assuming that no additional species are generated during the diffusion process and that mass conservation is satisfied, Equation (13) can be rewritten as Equation (14):

(14) $$\frac{\partial C}{\partial t}\;+\;\nabla J\;=\;0$$

If ∇*D* = 0, Equations (13) and (14) can be combined and rewritten as Equation (15):

(15) $$\frac{\partial C}{\partial t}\;=\;D\nabla^{2}C$$

By comparing the above formulations, the consistency between the constitutive relations of heat transfer and hydrogen diffusion indicates that both processes can be analyzed using the same computational framework. In other words, the hydrogen permeation behavior can be treated as an equivalent heat conduction problem, enabling an effective numerical simulation of hydrogen transport [41]. Accordingly, in this study, the hydrogen permeation behavior of the specialized PA12 material is modeled using the heat transfer module in Abaqus by invoking its built-in subroutines.

### 3.3. Models and Boundary Conditions

#### 3.3.1. Circular Specimens

**Figure 6:**
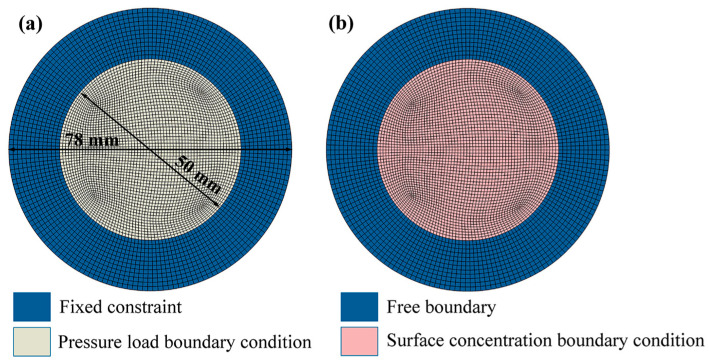
Finite element model of hydrogen permeation sample: (**a**) pressure load boundary condition; (**b**) Surface concentration boundary condition.

The geometric model of the test specimen was generated using a script of 3.12 version Python, and the corresponding material properties were assigned to the structure. The load and boundary conditions were then defined for different environmental scenarios. First, a coupled temperature–stress analysis was performed to obtain the stress distribution at various temperatures. In this step, room temperature was defined as 0 °C, and different temperature loads were applied using predefined field variables. Subsequently, the temperature-induced stress distributions were imported as initial conditions into the pressure–stress analysis model to evaluate the stress response under different combinations of temperature and pressure. Fixed constraints were applied along the annular surfaces with inner and outer diameters of 50 mm and 78 mm, respectively, while a uniformly distributed pressure was applied to a circular area with a diameter of 50 mm on the upper surface of the specimen, as illustrated in Figure 6a. Finally, the stress distributions obtained under various temperature and pressure conditions were used as initial conditions for the coupled stress–hydrogen permeation analysis. In the permeation model, Henry’s law [42] was employed to determine the hydrogen concentration C on the material surface under pressures of 7 MPa and 70 MPa.

(16) $$C=SP_{H}$$

Here, S denotes the solubility coefficient of hydrogen in the material (mol/m^3^·Pa), and $P_{H}$ represents the partial pressure of hydrogen on the circular region at the upper surface of the specimen (Pa). Meanwhile, the hydrogen concentration on the circular region with a diameter of 50 mm at the lower surface is prescribed as zero, as illustrated in Figure 6b. In the temperature–stress–pressure coupled analysis, the model is discretized using 27,315 C3D8R elements; for the stress–hydrogen diffusion coupled analysis, the element type is switched to DC3D20. The material parameters used in the finite element model are listed in Table 2.

#### 3.3.2. Type IV Hydrogen Storage Vessels

**Figure 7:**
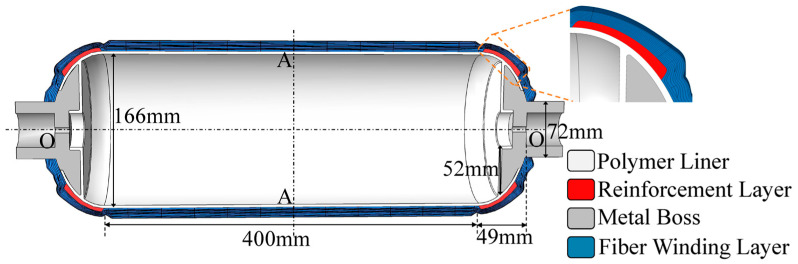
Geometric model of type IV hydrogen storage cylinder with a local reinforcement patch.

The filament-wound layer of the type IV hydrogen storage vessel consists of 22 hoop layers and 22 helical layers, with a stacking sequence of [90_6_/±21_2_/±33_2_/90_4_/±43_2_/90_4_/±21_2_/90_4_/±21_2_/90_4_/±21]. A schematic of the geometry of the type IV vessel with local reinforcement is shown in Figure 7.

**Figure 8:**
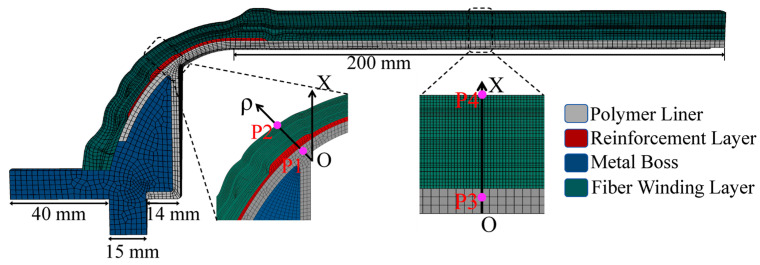
Finite element model of type IV hydrogen storage vessel with a local reinforcement patch.

Due to the symmetry of the type IV hydrogen storage vessel with respect to the neutral plane (A–A), half of the vessel was selected for numerical analysis. Furthermore, since the vessel also exhibits rotational symmetry about the central axis (O–O), computational cost was reduced by modeling a 10° sector extracted from the half model. Using ABAQUS, the liner, metallic boss, and locally reinforced regions of the vessel were generated through a Python script. Based on the outer contour formed by the liner, boss, and reinforcing layers, a 10° fiber-wound section of the vessel was created using the WCM module, followed by mesh generation to obtain the finite element model of the type IV vessel. The metallic boss, polymer liner, local reinforcement patch, and fiber winding layers were all modeled using DC3D20 elements, resulting in a total of 50,416 elements. The finite element model is shown in Figure 8. The polymer liner thickness is 3 mm, and the local reinforcement layer has a thickness of 1.2 mm.

To compare the hydrogen permeation behavior of different storage cylinders, the hydrogen mass flow rate and mass concentration at various locations (P1, P2, P3, and P4, as shown in Figure 8) were extracted and analyzed. Specifically, P1 and P3 are located in the dome and cylindrical regions, respectively, at two-thirds of the liner thickness measured from the inner surface. P2 and P4 correspond to nodes situated in the dome and cylindrical regions near the outermost part of the composite winding layer.

**Figure 9:**
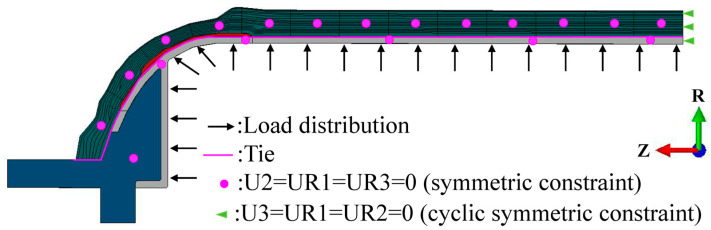
Boundary and load distribution map.

In the finite element analysis of the stress field of the type IV hydrogen storage vessel, a symmetric constraint in the Z-direction was applied on the axial symmetry plane of the vessel, while cyclic symmetric constraints in the circumferential direction were imposed on the radial cross-section. A uniformly distributed internal hydrogen working pressure was applied to the inner surface of the vessel. The mechanical properties of the type IV hydrogen storage vessel are listed in Table 3. For the hydrogen permeation analysis of the type IV vessel, a uniform pressure of 87.5 MPa was applied to the inner wall, whereas a zero hydrogen-concentration boundary condition was prescribed on the outer wall to drive hydrogen diffusion outward from the initial concentration at the inner surface. The hydrogen permeation parameters used for the type IV vessel are provided in Table 4. The loading configuration of the vessel is illustrated in Figure 9.

## 4. Results and Discussions

### 4.1. Assessment of Numerical Reliability

**Figure 10:**
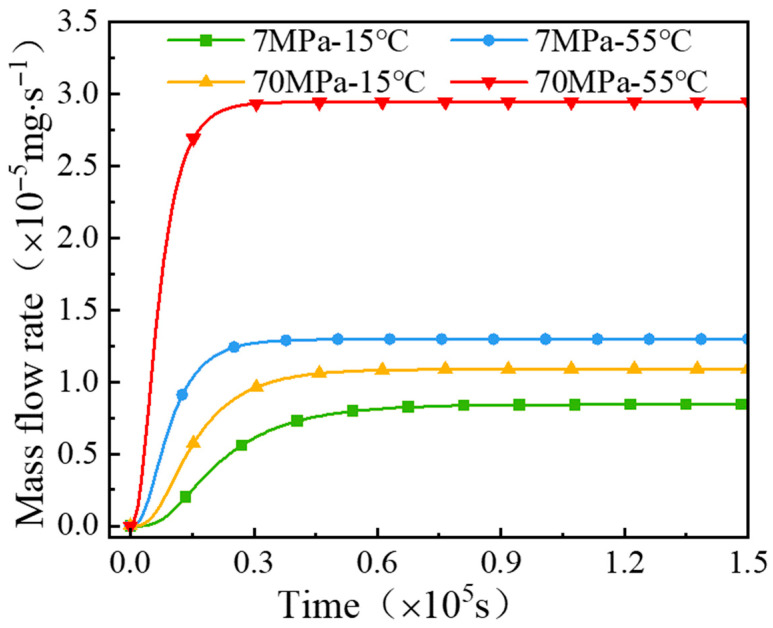
Mass flow rate curves on the outer side of PA12 under different operating conditions.

Figure 10 presents the hydrogen mass flow rate on the outer surface of the PA12 specimens under different test conditions. It can be seen that, the permeation reaches steady state most rapidly at 70 MPa and 55 °C, where the mass flow rate is also the highest. Once steady-state diffusion is established, the hydrogen mass flow rates on the outer surface of the specimens under the conditions of 7 Mpa-15 °C, 7 Mpa-55 °C, 70 Mpa-15 °C, and 70 Mpa-55 °C are 8.437 × 10^−6^ mg/s, 1.297 × 10^−5^ mg/s, 1.088 × 10^−5^ mg/s, and 2.944 × 10^−5^ mg/s, respectively.

According to the GB/T 42610-2023 standard, the mass flow rate of hydrogen on the outer side of the specimen under steady-state conditions can be expressed as:

(17) $${\frac{\Delta Q}{\Delta t}}_{e}=\;\frac{P_{e}\cdot S\cdot \Delta p}{B}$$

here, $P_{e}$ is the gas permeability coefficient of the material (mol⋅m/(m^2^⋅s⋅Pa)); B is the thickness of the disk-shaped specimen (m). S is the surface area of the specimen exposed to hydrogen during the permeation test (m^2^). Δp is the pressure difference between the high-pressure and low-pressure sides of the specimen during the test (Pa). Based on the data in Table 2 and Equation (17), the hydrogen transmission rate under steady-state conditions can be calculated.

**Figure 11:**
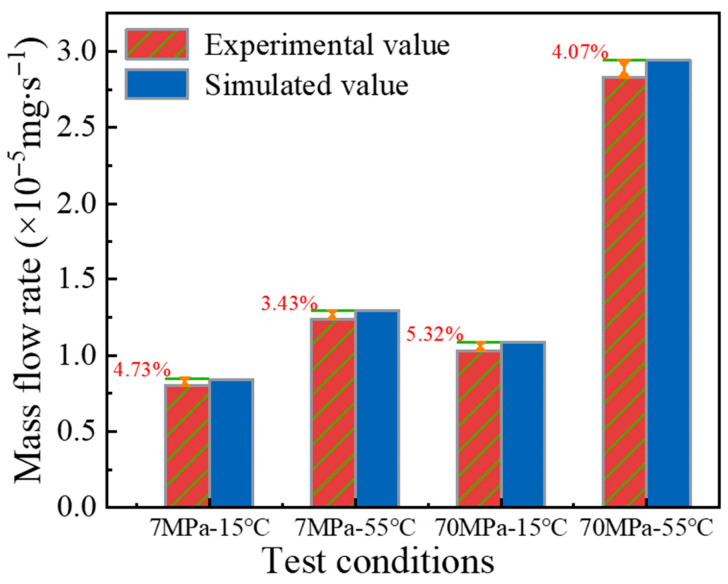
Comparison of experimental and numerical mass flow rates of hydrogen permeation under steady-state conditions.

The comparison between the experimental and numerical mass flow rates at steady-state hydrogen permeation is shown in Figure 11. The good agreement between the experimental measurements and numerical predictions verifies the reliability of the numerical analysis approach. The discrepancy between the experimental and numerical results is approximately 5%. At the same temperature, the deviation increases with increasing pressure. This trend is mainly attributed to material densification under higher pressure, which leads to subtle changes in its mechanical properties.

**Figure 12:**
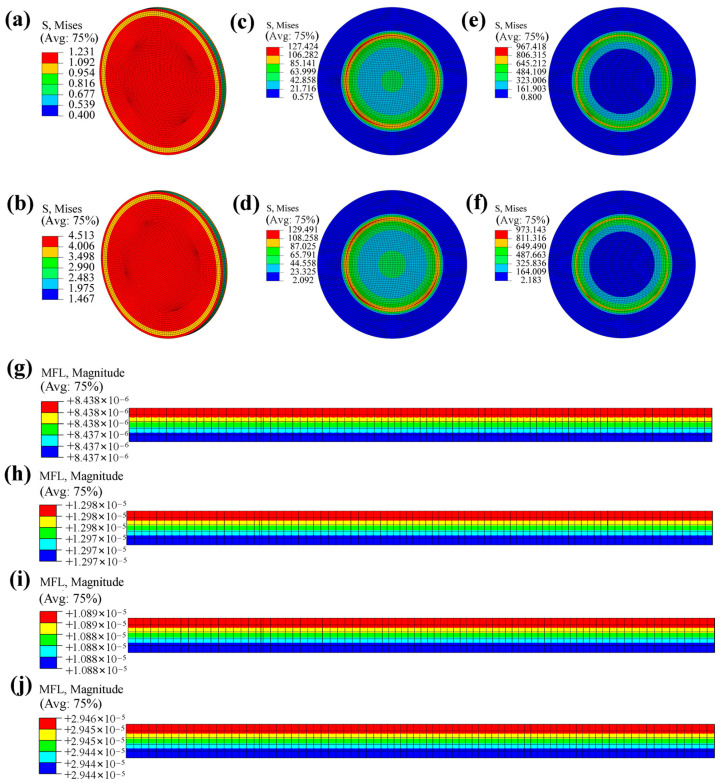
Numerical results of hydrogen permeation in PA12 sample: (**a**) Thermal stress at 15 °C; (**b**) Thermal stress at 55 °C; (**c**) Stress at 7 MPa and 15 °C; (**d**) Stress at 7 MPa and 55 °C; (**e**) Stress at 70 MPa and 15 °C; (**f**) Stress at 70 MPa and 55 °C; (**g**) Mass flow rate at 7 MPa and 15 °C; (**h**) Mass flow rate at 7 MPa and 55 °C; (**i**) Mass flow rate at 70 MPa and 15 °C; (**j**) Mass flow rate at 70 MPa and 55 °C.

Figure 12 presents the contour plots of the multi-field coupled numerical analyses under different operating conditions. The comparison indicates that, at a given pressure, the stress distributions of the specimens at 15 °C and 55 °C differ only slightly. In contrast, at a fixed temperature, the stresses corresponding to 7 MPa and 70 MPa vary significantly, and higher pressures lead to a larger hydrogen mass flux on the outer surface of the specimens.

Furthermore, under steady-state diffusion, the hydrogen concentration differences between the front and back surfaces are 0.01 × 10^−7^, 0.01 × 10^−6^, 0.01 × 10^−6^, and 0.02 × 10^−6^ for the conditions of 7 MPa-15 °C, 7 MPa-55 °C, 70 MPa-15 °C, and 70 MPa-55 °C, respectively. Therefore, although the spatial distributions of hydrogen concentration within the material remain generally similar across different operating conditions, their concentration gradients exhibit pronounced differences.

### 4.2. Hydrogen Permeation Behavior of Hydrogen Storage Vessels

#### 4.2.1. Influence of Local Reinforcement on Hydrogen Permeation Behavior

The transport of hydrogen within the constituent materials of the cylinder structure follows Fick’s law [51]. In the initial stage, the chemical potential difference between the inner and outer surfaces generates a steep concentration gradient. According to transition-state diffusion theory, hydrogen molecules rapidly overcome the energy barrier and diffuse inward under this high gradient. As diffusion progresses, hydrogen migrates toward the outer layers, where scattering at grain boundaries and other micro-structural features increases the diffusion resistance with depth [52]. Consequently, the diffusion flux decreases and the concentration gradient gradually becomes less pronounced.

Moreover, under internal pressure load, the composite winding layers will undergo deformation. In hydrogen storage vessels with local reinforcement, the reinforcement patches work synergistically with the winding layer fibers in the dome section to carry the load, thereby reducing stress concentrations in the dome region and extending the hydrogen diffusion path [53,54].

**Figure 13:**
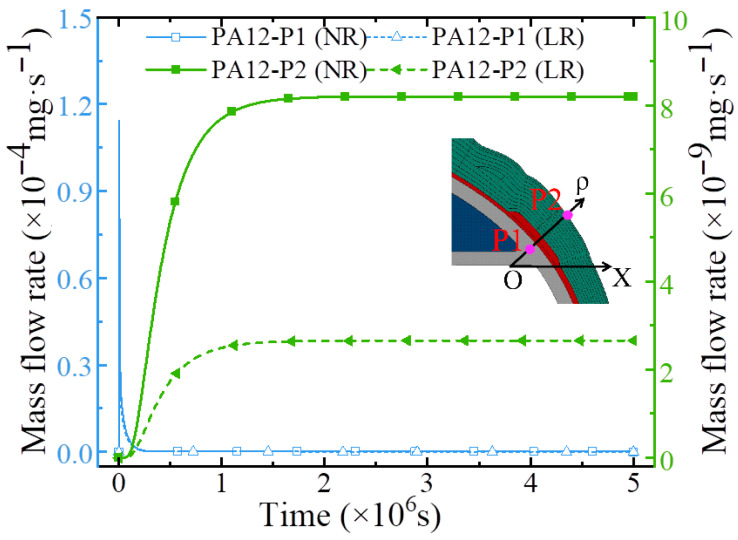
The mass flow rate of dome region of hydrogen storage cylinder with a local reinforcement patch.

Using PA12 at 70 MPa and 55 °C as the liner material, the influence of local reinforcement on the hydrogen permeation behavior of the vessel structure was investigated. Figure 13 compares the permeation results of type IV hydrogen storage vessels with local reinforcement (LR) and without local reinforcement (NR). As shown in Figure 13, the presence of local reinforcement has no significant effect on the mass flow rate at point P1. When hydrogen permeation reaches steady state, the mass flow rates at P1 are nearly identical for both the LR and NR vessels. However, during the entire diffusion process, from the initial stage to steady state, the mass flow rate at point P2 in the LR vessel remains consistently lower than that in the NR vessel. At steady state, the mass flow rate at P2 in the NR vessel is 8.201 × 10^−9^ mg/s. Compared with the NR vessel, the LR vessel exhibits a 67.6% reduction in the mass flow rate at P2.

**Figure 14:**
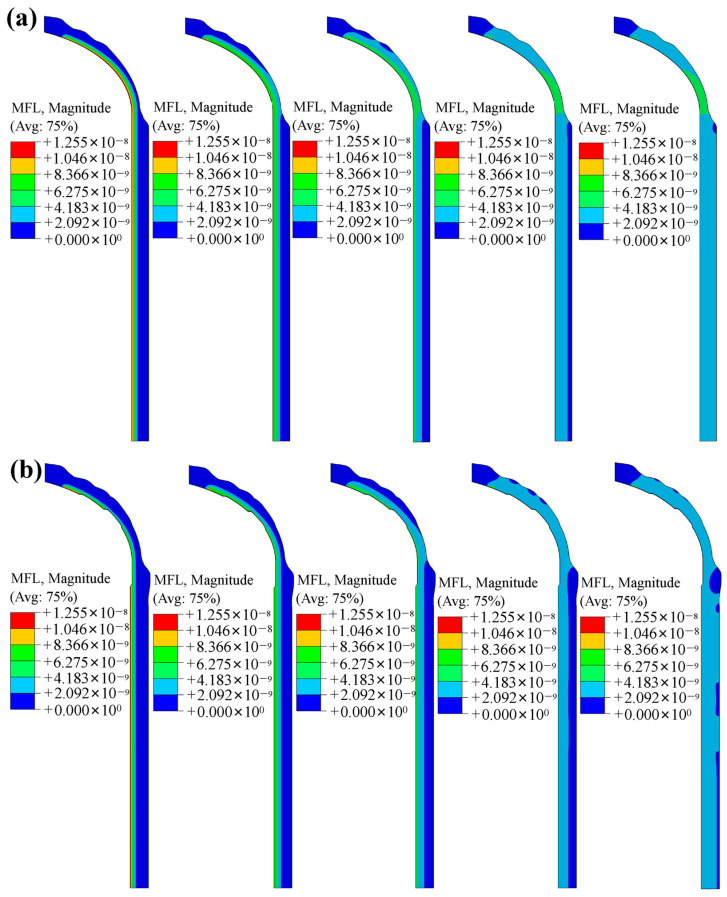
Mass flow rate of fiber winding layer of hydrogen storage vessel with the PA12 liner: (**a**) without the reinforcement patch; (**b**) with the reinforcement patch.

Figure 14 presents the mass flow rate contours of the winding layer in the LR and NR hydrogen storage vessels at different time instants. Comparative analysis shows that the local reinforcement has a pronounced effect on the mass flow rate in the dome region, while its influence on the cylindrical section is minimal. Compared with the NR vessel, the LR vessel exhibits a lower mass-flow rate in the transition region between the dome and the cylinder.

#### 4.2.2. Hydrogen Permeation Characteristics of Hydrogen Storage Vessels with Different Liner Materials

**Figure 15:**
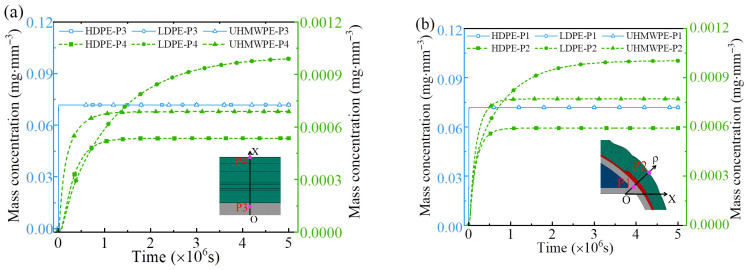
The mass concentration curves of hydrogen at different regions of the hydrogen storage vessel at different times: (**a**) cylindrical section; (**b**) dome section.

**Figure 16:**
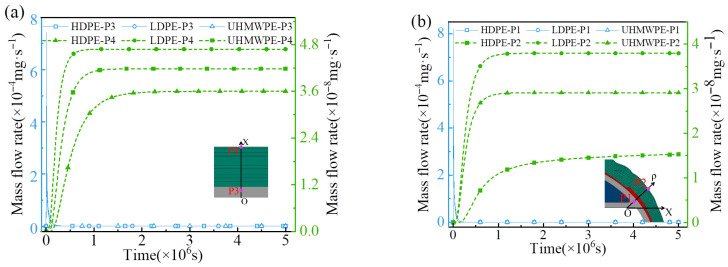
The mass flow rate curves of hydrogen at different regions of the hydrogen storage vessel at different times: (**a**) cylindrical section; (**b**) dome section.

Figure 15 and Figure 16 present the numerical hydrogen permeation curves of LR hydrogen storage vessels with different liner materials. At the initial stage of hydrogen diffusion, the polymer liner, acting as a single material layer, allows hydrogen molecules to first dissolve into the free-volume cavities between polymer chains [55]. Subsequently, driven by the concentration gradient, the dissolved molecules migrate outward through thermally activated diffusion. As time progresses, the hydrogen concentration on the outer side of the liner gradually increases, leading to a reduction in the concentration gradient and, consequently, a continuous decrease in the diffusion rate within the liner [56]. Meanwhile, due to the continuous ingress of hydrogen molecules, both the mass flow rate and mass concentration in the external composite winding layer progressively increase.

Throughout the hydrogen permeation process, the hydrogen mass concentration and mass flow rate at points P1 and P3 remain identical for all liner materials. However, once hydrogen permeation reaches steady state, significant differences arise at points P2 and P4, which are located near the outer surface of the vessel. Among the examined liners, HDPE exhibits the lowest hydrogen diffusion coefficient and solubility, resulting in the smallest hydrogen mass concentration and mass flow rate at the outer surface (P2 and P4) of the LR vessel under steady-state conditions. In contrast, the LDPE and UHMWPE liners possess higher diffusion coefficients and solubilities. Consequently, at steady state, LR vessels with LDPE or UHMWPE liners show higher hydrogen mass concentrations and mass flow rates at the outer surface (P2 and P4).

**Figure 17:**
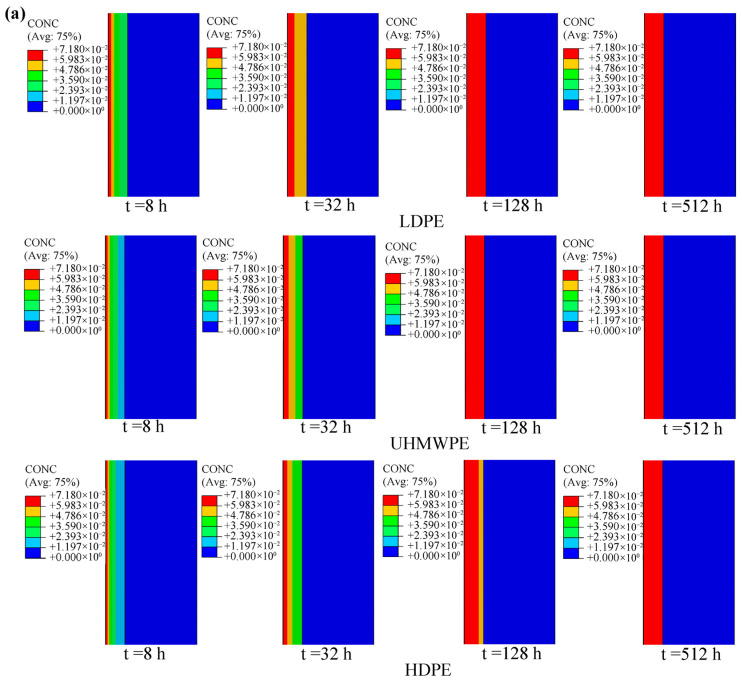

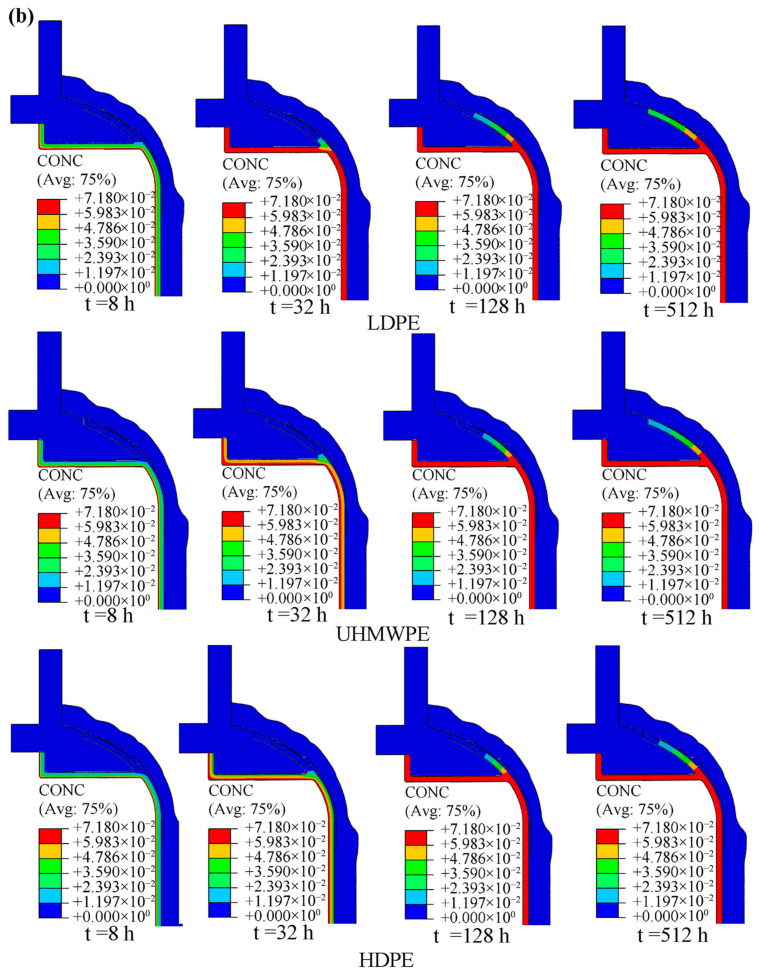
Mass concentration at different regions of the hydrogen storage vessel at different times: (**a**) cylindrical section; (**b**) dome section.

**Figure 18:**
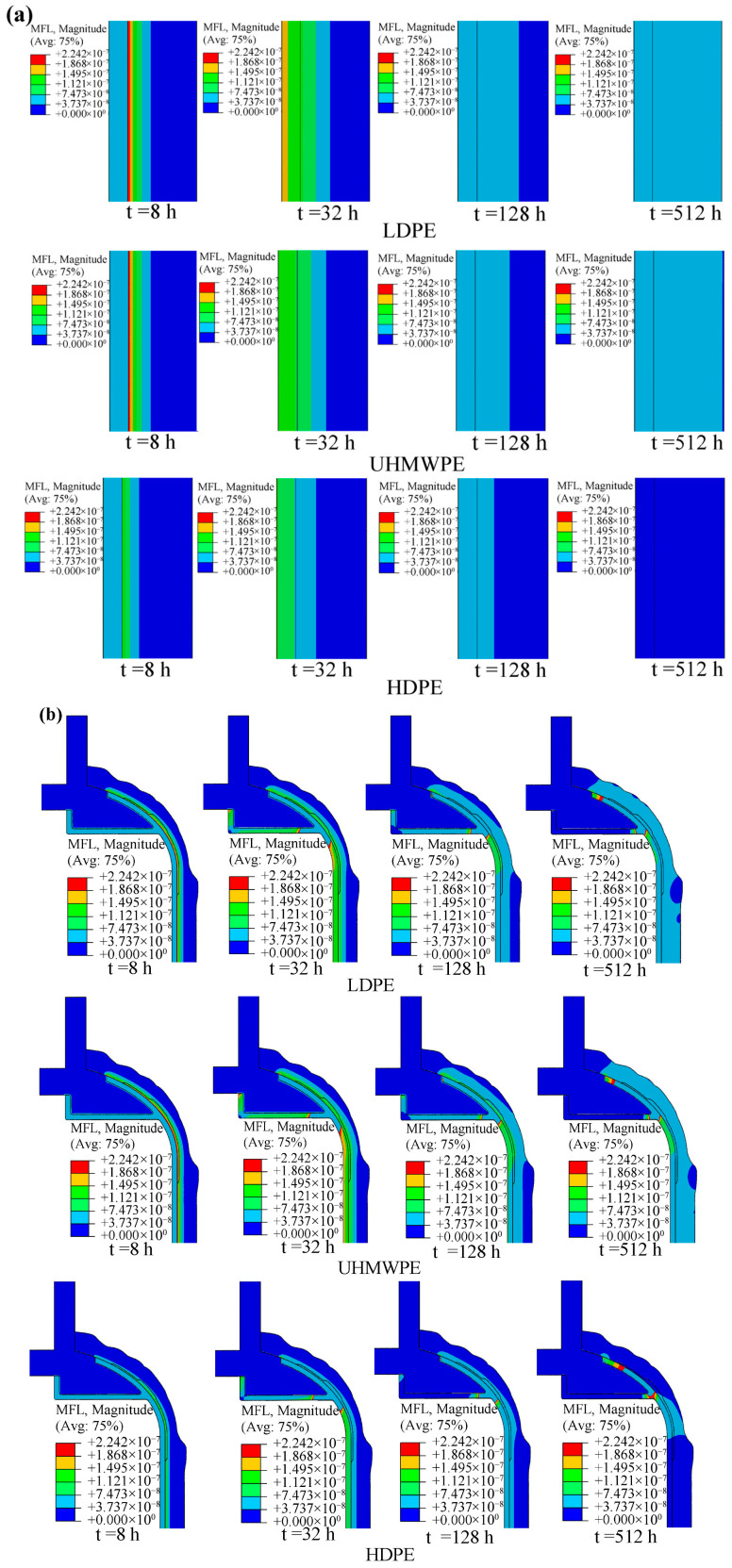
Mass flow rate of hydrogen at different areas of the hydrogen storage vessel at different times: (**a**) cylindrical section; (**b**) dome section.

Figure 17 and Figure 18 present the hydrogen permeation contours of LR hydrogen storage vessels with different liner materials. Before reaching steady state (e.g., t = 8 h), the distribution of hydrogen mass concentration within the liners differs among vessels with different liner materials. A lower hydrogen diffusion coefficient in the liner leads to lower hydrogen mass concentration and mass flux within the liner.

At steady state, the mass flux and mass concentration in the composite winding layer are significantly lower than those in the liner. This is attributed to the fact that the composite winding layer is composed of fibers and resin, where densely packed fiber bundles with high volume fraction are tightly interlaced with the resin matrix [57]. In addition, the fiber–matrix interfaces exhibit strong adsorption effects on hydrogen molecules [58], further inhibiting permeation.

In the dome region of the vessel, two factors contribute to locally elevated mass flux. First, the large local curvature increases the probability of molecular collisions, intensifying hydrogen diffusion [59]. Second, under internal pressure, the dome region experiences higher local stresses. The stress field induced by pressure enhances hydrogen diffusion, and this enhancement becomes more pronounced as stress increases [60]. Consequently, during steady-state diffusion, the dome region exhibits higher local mass flux, as shown in Figure 18.

#### 4.2.3. Hydrogen Permeation Mass of the Hydrogen Storage Vessel

Based on the time-dependent mass flow rate curves measured at different locations on the outer surface of the hydrogen storage vessel, the total hydrogen permeation before and after reaching steady state was obtained by integrating the flow rate over time. The hydrogen permeation is calculated using the following equation:

(18) $$Q\;=\int_{t_{1}}^{t_{2}}\int_{0}^{A}M\;(t)\mathrm{dAdt}$$

Here, Q is hydrogen permeation mass of hydrogen storage vessel. M(t) represents the mass flow rate at the outer surface of the vessel at the t moment. A is the outer surface area of the vessel. Based on the geometric parameters of the simulation model presented in Figure 7, the external surface area of the hydrogen storage cylinder is calculated to be 0.45 m^2^. t_1_ and t_2_ denote the time interval before and after the hydrogen permeation measurement.

**Figure 19:**
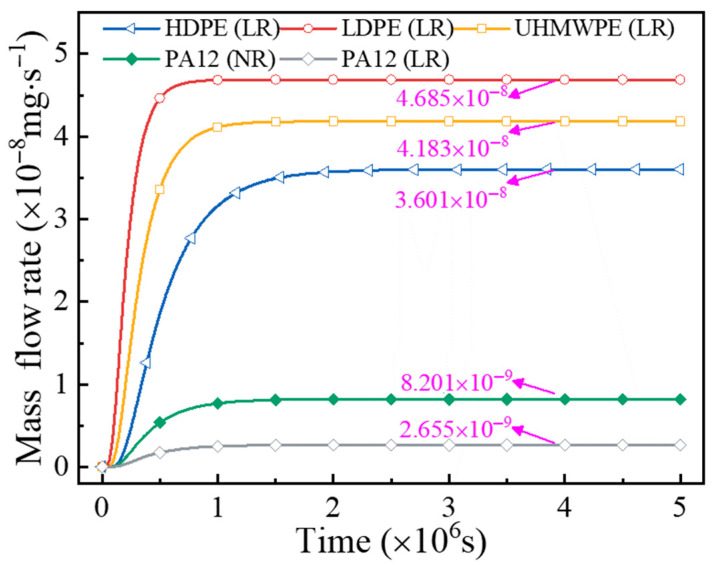
Mass flow rate curves of the outer surfaces of hydrogen storage vessel with different liners.

The mass flow rates at positions P1 and P2 of the same hydrogen storage vessel exhibit no significant difference compared to that of the cylindrical section, as shown in Figure 16. Therefore, the hydrogen permeation amount prior to reaching steady state is quantified based on the time-dependent mass flow rate on the outer surface of the cylinder. Figure 19 presents the mass flow rate curves at the outer-surface nodes for hydrogen storage vessels equipped with different liners. When hydrogen diffusion reaches steady state, the mass flow rates on the outer surface of LR hydrogen storage vessels with liners made of HDPE, LDPE, UHMWPE, and PA12 are 3.601 × 10^−8^ mg/s, 4.685 × 10^−8^ mg/s, 4.183 × 10^−8^ mg/s, and 2.655 × 10^−9^ mg/s, respectively. For vessels using PA12 as the liner material, the mass flow rate on the outer surface of the NR hydrogen storage vessel increases by 208.89% compared with that of the locally reinforced vessel, reaching 8.201 × 10^−9^ mg/s.

The mass flow rate–time curves were fitted using an appropriate function to obtain the hydrogen mass flow rate function M(t) on the external surface of the cylinder at different time points, expressed as:

(19) $$M(t)\;={\;M}_{\mathrm{ss}}\cdot (1-e^{-\mathrm{kt}})$$

Here, $M_{\mathrm{SS}}$ represents the mass flow rate at steady state. k is the apparent time constant characterizing the rate of approach to steady state. The fitting yielded a coefficient of determination R^2^ > 0.99, indicating an excellent agreement between the model and the experimental data.

By combining Equations (18) and (19), the total hydrogen permeation volumes of the whole cylinder before reaching steady state and within one hour after reaching steady state are quantitatively calculated. The results are presented in Table 5. It can be clearly observed that the higher the material’s diffusion coefficient, the shorter the time required for the hydrogen diffusion behavior of the storage cylinder to reach steady state, while the total amount of hydrogen permeation before and after reaching steady state increases. Furthermore, compared with the NR hydrogen storage vessel, local reinforcement extends the time required for the hydrogen diffusion behavior to reach steady state, but significantly reduces the total hydrogen permeation both before and after steady state. Specifically, during the previous steady period and the first hour after reaching steady state, the total hydrogen permeation of the NR hydrogen storage vessel was 1.041 × 10^4^ mg and 13.286 mg, respectively. In contrast, for the LR hydrogen storage vessel, the corresponding hydrogen permeation decreased by 68.84% and 67.62%, respectively. Therefore, the locally reinforced structure not only enhances the load-bearing capacity of the dome region but also effectively improves the overall hydrogen permeation behavior of the storage cylinder.

In addition, the hydrogen permeation limit specified in the ISO 19881 standard [61] is ≤46 NmL/(h·L). Therefore, the calculated results indicate that cylinders using HDPE, LDPE, or UHMWPE as the inner liner material fail to meet the required hydrogen permeation standard, whereas the use of PA12 as the inner liner material satisfies the specification.

## 5. Conclusions

In this study, a combined experimental and numerical approach was employed to investigate the hydrogen permeation characteristics of specialized PA12 materials under different temperature and pressure conditions. Based on a validated finite element numerical analysis method, the hydrogen permeation behavior of type IV hydrogen storage cylinders with different liner materials was further analyzed. The main findings are summarized as follows:

Temperature and pressure have significant effects on the hydrogen permeation properties of PA12. At 15 °C and 55 °C, when the pressure increased from 7 MPa to 70 MPa, the hydrogen permeability of the material decreased by 87.18% and 77.23%, respectively. In addition, when the temperature increased from 15 °C to 55 °C, the hydrogen permeability increased by 54.20% at 7 MPa and 173.81% at 70 MPa.

During steady-state diffusion, the hydrogen mass flux at the outer surface of the PA12 specimens agreed well with the numerical predictions, demonstrating the reliability of the coupled multi-field numerical analysis method. The numerical results indicate that, although the spatial distribution of hydrogen concentration within the material is generally consistent under different temperature and pressure conditions, the concentration gradients vary significantly.

Numerical analysis of hydrogen permeation in the storage cylinders shows that local reinforcement strategies can suppress hydrogen permeation rates in specific regions and enhance the overall hydrogen barrier performance, thereby reducing the hydrogen permeation before and after reaching steady state. Furthermore, as the diffusion coefficient of the liner material increases, the hydrogen diffusion rate inside the storage cylinder significantly rises, resulting in a shorter time required to reach steady-state permeation.

These findings expand the database and understanding of the permeation behavior of PA12 material under high-pressure hydrogen, while providing guidance for optimizing material selection, localized reinforcement, and overall hydrogen permeation control strategies in the type IV hydrogen storage vessel.

## Figures and Tables

**Table 1:** Hydrogen permeation parameters of PA12 materials at different temperatures and pressures.

Cases	P (mol·m·m^−2^·s^−1^·Pa^−1^)	D (m^2^·s^−1^)	S (mol·m^−3^·Pa^−1^)
7 MPa-15 °C	(1.31 ± 0.31) × 10^−15^	(1.41 ± 0.24) × 10^−10^	(9.29 ± 0.82) × 10^−6^
7 MPa-55 °C	(2.02 ± 0.35) × 10^−15^	(3.35 ± 0.27) × 10^−10^	(6.03 ± 0.82) × 10^−6^
70 MPa-15 °C	(1.68 ± 0.22) × 10^−16^	(2.02 ± 0.12) × 10^−10^	(8.36 ± 1.16) × 10^−7^
70 MPa-55 °C	(4.60 ± 0.27) × 10^−16^	(4.68 ± 0.09) × 10^−10^	(9.90 ± 1.38) × 10^−7^

Detailed data can be found in Appendix Table A1.

**Table 2:** Material properties and parameters of the PA12.

Material Properties		Values
Elastic modulus (MPa)	15 °C	1300 [43]
	55 °C	900 [43]
Poisson’s ratio	15 °C	0.33 [43]
	55 °C	0.37 [43]
Thermal conductivity coefficient (W·m^−1^·K^−1^)		0.5 [44]
Thermal expansion coefficient (cm·cm^−1^·°C^−1^)		3.8 × 10^−5^ [44]
Specific heat capacity (J·kg^−1^·K^−1^)		1640 [44]
Density (g·cm^−3^)		1.08 × 10^−6^ [45]

**Table 3:** Performance parameters of type IV hydrogen storage cylinders materials.

Materials	Density (g·cm^−3^)	Elastic Modulus (MPa)	Poisson’s Ratio
HDPE [46]	0.948	1300	0.38
LDPE [46]	0.920	540	0.394
UHMWPE [47]	0.936	820	0.46
Al [48]	2.7	69,000	0.324
CFRP [49]	1.8	154.1	0.35

**Table 4:** Finite element model material parameters.

Materials	Diffusion Coefficient (m^2^·s^−1^)	Solubility Coefficient (mol·m^−3^·Pa^−1^)
LDPE [23]	15.1 × 10^−10^	9.68 × 10^−7^
HDPE [23]	2.3 × 10^−10^	2.17 × 10^−6^
UHMWPE [23]	8.8 × 10^−10^	1.01 × 10^−6^
Al [50]	11.9 × 10^−15^	9.73 × 10^−9^
CFRP [37]	4.5 × 10^−13^	2.32 × 10^−9^

**Table 5:** The hydrogen permeability of gas cylinders with different inner lining materials before and 1 h after steady state.

Inner Lining Material	Reach the Steady-State Time (d)	Total Hydrogen Permeation Before Steady State (mg)	Hydrogen Permeation Volume 1 h After Steady State (mg)
PA12 (NR)	38.08	1.041 × 10^4^	13.286
PA12 (LR)	40.86	0.366 × 10^4^	4.302
HDPE (LR)	36.34	2.061 × 10^4^	58.337
UHMWPE (LR)	26.74	3.676 × 10^4^	67.766
LDPE (LR)	14.01	4.139 × 10^4^	75.910

## Data Availability

The data that support the findings of this study are available from the corresponding author upon reasonable request.

## Abbreviations

The following abbreviations are used in this manuscript:

PA12	Polyamide 12
CFRP	Carbon fiber-reinforced resin-based plastic
Al	Aluminum
DMA	Dynamic thermomechanical analysis
PE	Polyethylene
HDPE	High density polyethylene
LDPE	Low density polyethylene
UHMWPE	Ultra-high molecular weight polyethylene
DR	Dome reinforcement
PA11	Polyamide 11
PA6	Polyamide 6
FEA	Finite element analysis
LR	Local reinforcement
NR	No reinforcement

## Appendix A

**Table A1:** Hydrogen permeation test data under different temperatures and pressures.

Cases	Sample Number	P (mol·m·m^−2^·s^−1^·Pa^−1^)	D (m^2^·s^−1^)	S (mol·m^−3^·Pa^−1^)
7 MPa-15 °C	1	1.12 × 10^−15^	1.27 × 10^−10^	8.69 × 10^−6^
	2	1.05 × 10^−15^	1.73 × 10^−10^	10.69 × 10^−6^
	3	1.56 × 10^−15^	1.51 ×10^−10^	9.35 × 10^−6^
	4	1.09 × 10^−15^	1.44 × 10^−10^	8.92 × 10^−6^
	5	1.73 × 10^−15^	1.10 × 10^−10^	8.80 × 10^−6^
Average value		1.31 × 10^−15^	1.41 × 10^−10^	9.29 × 10^−6^
Sample standard deviation		0.31 × 10^−15^	0.24 × 10^−10^	0.82 × 10^−6^
7 MPa-55 °C	1	2.12 × 10^−15^	3.42 × 10^−10^	5.02 × 10^−6^
	2	2.00 × 10^−15^	3.16 × 10^−10^	6.14 × 10^−6^
	3	2.56 × 10^−15^	3.78 × 10^−10^	5.78 × 10^−6^
	4	1.69 × 10^−15^	3.28 × 10^−10^	5.92 × 10^−6^
	5	1.73 × 10^−15^	3.11 × 10^−10^	7.29 × 10^−6^
Average value		2.02 × 10^−15^	3.35 × 10^−10^	6.03 × 10^−6^
Sample standard deviation		0.35 × 10^−15^	0.27 × 10^−10^	0.82 × 10^−6^
70 MPa-15 °C	1	1.37 × 10^−16^	2.07 × 10^−10^	6.62 × 10^−7^
	2	1.94 × 10^−16^	1.95 × 10^−10^	9.58 × 10^−7^
	3	1.82 × 10^−16^	2.18 × 10^−10^	8.91 × 10^−7^
	4	1.69 × 10^−16^	1.86 × 10^−10^	8.89 × 10^−7^
	5	1.58 × 10^−16^	2.04 × 10^−10^	7.80 × 10^−7^
Average value		1.68 × 10^−16^	2.02 × 10^−10^	8.36 × 10^−7^
Sample standard deviation		0.22 × 10^−16^	0.12 × 10^−10^	1.16 × 10^−7^
70 MPa-55 °C	1	4.91 × 10^−16^	4.47 × 10^−10^	1.21 × 10^−6^
	2	5.12 × 10^−16^	4.87 × 10^−10^	1.01 × 10^−6^
	3	3.84 × 10^−16^	4.63 × 10^−10^	8.40 × 10^−7^
	4	4.05 × 10^−16^	4.51 × 10^−10^	9.23 × 10^−7^
	5	5.08 × 10^−16^	4.92 × 10^−10^	9.67 × 10^−7^
Average value		4.60 × 10^−16^	4.68 × 10^−10^	9.90 × 10^−7^
Sample standard deviation		0.27 × 10^−16^	0.09 × 10^−10^	1.38 × 10^−7^
